# The Effect of Training Interventions on Change of Direction Biomechanics Associated with Increased Anterior Cruciate Ligament Loading: A Scoping Review

**DOI:** 10.1007/s40279-019-01171-0

**Published:** 2019-09-06

**Authors:** Thomas Dos’Santos, Christopher Thomas, Paul Comfort, Paul A. Jones

**Affiliations:** Human Performance Laboratory, University of Salford, Greater Manchester, UK

## Abstract

Change of direction (COD) manoeuvres are associated with anterior cruciate ligament (ACL) injury risk due to the propensity to generate large multiplanar knee joint loads. Given the short- and long-term consequences of ACL injury, practitioners are interested in methods that reduce knee joint loads and subsequent ACL loading. An effective strategy to reduce ACL loading is modifying an athlete’s movement mechanics to reduce knee joint loading. The purpose of this scoping review was to critically appraise and comprehensively synthesise the existing literature related to the effects of training interventions on COD biomechanics associated with increased knee joint loads and subsequent ACL loading, and identify gaps and recommend areas for future research. A review of the literature was conducted using Medline and Sport DISCUS databases. Inclusion criteria consisted of pre-post analysis of a COD task, a minimum 4-week training intervention, and assessments of biomechanical characteristics associated with increased ACL loading. Of the 1,027 articles identified, 22 were included in the scoping review. Based on current literature, balance training and COD technique modification are the most effective training modalities for reducing knee joint loading (small to moderate effect sizes). One study reported dynamic core stability training was effective in reducing knee joint loads, but further research is needed to definitively confirm the efficacy of this method. Perturbation-enhanced plyometric training, the F-MARC 11 + soccer specific warm-up, Oslo Neuromuscular warm-up, and resistance training are ineffective training modalities to reduce COD knee joint loads. Conflicting findings have been observed for the Core-Pac and mixed training programme. Consequently, practitioners should consider incorporating balance and COD technique modification drills into their athletes’ training programmes to reduce potentially hazardous knee joint loads when changing direction. However, training intervention studies can be improved by investigating larger sample sizes (> 20), including a control group, acknowledging measurement error when interpreting their findings, and considering performance implications, to confirm the effectiveness of training interventions and improve adherence.

## Key Points

**Table:** 

Modifying an athlete’s change of direction mechanics by addressing biomechanical and neuromuscular deficits associated with hazardous knee joint loading is an effective strategy to reduce anterior cruciate ligament loading. This can be achieved through biomechanical and neuromuscular informed training interventions.
Balance training is a potentially effective strategy to reduce knee joint loads during cutting, most likely attributed to eliciting safer knee agonist-antagonist muscle patterns and hip and trunk muscle activity. Further research is necessary in greater sample sizes and acknowledging measurement error when interpreting findings, to definitively confirm the efficacy of this method.
Change of direction technique modifications that focus on reducing lateral trunk flexion, reducing lateral foot plant distances, increasing knee flexion, and promoting earlier braking (during the penultimate foot contact), provide an effective training modality for reducing COD knee joint loading. However, in order to confirm the efficacy and adherence of this method, studies can be improved by including a control group, investigating larger sample sizes, acknowledging measurement error when interpreting findings, and considering the performance implications.

## Introduction

Anterior cruciate ligament (ACL) injury is a serious, debilitating injury with short- and long-term consequences (financial, health and psychological) [1–5], with an elevated and earlier risk of developing osteoarthritis a primary concern [4, 6]. Annual ACL injury rates are estimated to be 250,000 in the USA [1] and two million injuries worldwide [7], with in excess of US$1 billion estimated to be spent annually on reconstruction and rehabilitation in the USA. Anterior cruciate ligament injuries typically require surgery when athletes wish to return to cutting-based sports [8]; thus, extensive rehabilitation periods are required, resulting in prolonged absence and the potential to lose sporting scholarships or contracts [9]. Furthermore, athletes who do successfully return to sport post ACL reconstruction may demonstrate reduced sports-related performance (i.e. goals, shots per match, pass success, etc.), reduced number of appearances and minutes per match, and shorter career longevity [10–12]. Therefore, reducing the relative risk of ACL injury is of primary importance in sports medicine and strength and conditioning.

Anterior cruciate ligament injuries occur when a load is applied that exceeds the ligament’s tolerance threshold [13, 14]. Although ACL injury-risk factors are multifactorial (i.e. hormonal, anatomical, biomechanical and neuromuscular) [1, 15], a large proportion of ACL injuries in sports such as handball (60%) [16], American football (60%) [17] and rugby (67%) [18] occur during non-contact change of direction (COD) manoeuvres (cutting, pivoting, plant-and-cut actions). This occurrence can be attributed to the propensity to generate high forces and multiplanar knee joint loading (sagittal, frontal and transverse plane moments) during the plant foot contact when changing direction [19–23], thus increasing ACL strain [24–28]. For example, COD techniques with greater ground reaction forces (GRF) [20, 29, 30], lateral foot plant distance [21, 23, 31, 32], lateral trunk flexion over the plant foot [21, 31, 33, 34], hip abduction [29], internal foot progression (initial posture) [29, 35], hip internal rotation (initial posture) [29, 30, 32, 36] and peak knee abduction angles (KAA) [23, 30, 31, 35, 36] are associated with greater peak knee abduction moments (KAM), and thus ACL loading and potential injury risk [25, 37–41]. Additionally, wide lateral foot plant distances, trunk rotation towards the stance limb, trunk flexion displacements and hip internal rotation moments have been reported to be associated with greater knee internal rotation moments (IRMs) [21, 34], which when combined with KAMs (multiplanar) produces greater strain on the ACL compared to uniplanar loading [24–28]. Moreover, observational analysis of ACL injuries has also confirmed these kinematics as characteristics of non-contact injury during COD manoeuvres [16–18, 42–46]. Therefore, minimising and avoiding these potentially hazardous kinematic postures could be a viable strategy to reduce ACL loading and the relative risk of non-contact ACL injury during COD actions [41, 47, 48].

In order to reduce ACL loading and potential injury-risk during directional changes, particularly non-contact ACL injuries, an effective strategy is to modify an athlete’s movement mechanics by addressing biomechanical and neuromuscular deficits. This can be done through biomechanical and neuromuscular informed training interventions to reduce the magnitude of knee joint loading [1, 14, 41, 49–55]. Due to the prevalence of non-contact ACL injuries associated with COD actions in multidirectional sport [16–18, 42–46], various training interventions have been performed in an attempt to alter COD biomechanical characteristics associated with increased ACL loading. These include COD technique modification drills [22, 56], COD speed and footwork [57], mixed training programmes (sessions that integrate exercises from several training modalities, e.g. plyometrics, stretching, balance, trunk stabilisation and/or resistance training) [53, 58–61], combined trunk stabilisation and resistance training [62], resistance training [62, 63], combined COD technique modification drills and balance training [64], combined resistance training and intersegmental control training during running and COD drills [65], dynamic core stability training [66], balance training [63, 67, 68], perturbation-enhanced plyometric training [69], and injury-prevention warm-up protocols (i.e. Oslo, Core-Pac, F-MARC 11+) [53, 59, 70–75]. As practitioners working in multidirectional sports are interested in injury-risk mitigation strategies, understanding the most effective training modalities that address COD biomechanics associated with increased ACL loading is of great importance. The purpose of this scoping review was threefold: (1) to critically appraise and comprehensively synthesise the existing literature related to the effects of training interventions on COD biomechanics associated with increased knee joint loads and subsequent ACL loading; (2) to identify gaps in the literature and recommend areas for future research; and (3) to provide evidence-based recommendations that outline efficacious strategies for addressing COD biomechanics associated with increased ACL loading and potential non-contact injury risk.

## Literature Search Methodology

**Fig. 1:**
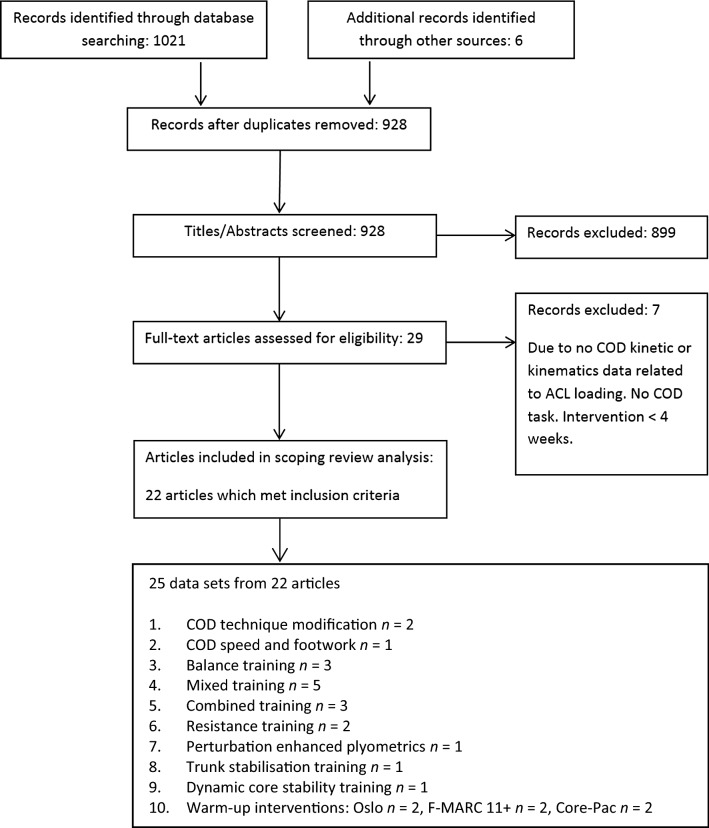
Flow diagram illustrating the different phases of the scoping review; based on PRISMA recommendations. *COD* change of direction, *ACL* anterior cruciate ligament

A literature search was performed using Medline and Sport Discus databases. Figure 1 provides a schematic representation of the search methodology in accordance with the PRISMA guidelines [76]. Search terms were as follows: (1) “biomechanics”, or “neuromuscular”, or “electromyography”, AND (2) “change of direction”, or “cutting”, or “cut”, or “sidestep”, or “turning”, AND (3); “intervention”, or “program”, or “programme”, or “training”, or “modification”. Bibliographies of relevant studies were hand searched to identify any additional studies. Citation tracking on Google Scholar was also used to identify any additional material. The search date ranged from 15 August 2018 to 10 January 2019. Articles were included for review if they met the following criteria:

1. Investigated a cutting or turning task (e.g. side-step, plant-and-cut actions, pivot).
2. Examined the effects of a training modality intervention (minimum 4 weeks) on COD biomechanics associated with increased ACL loading (e.g. knee valgus angle, knee abduction moments, knee flexion angle, knee rotation moments, knee flexion moment, vertical and posterior GRF, muscle activation, lateral trunk flexion, trunk rotation, foot progression angle, etc.).
3. Included participants who participated in sport or physical activity.

Studies that failed to meet the abovementioned criteria were subsequently excluded. Training intervention studies that met the abovementioned criteria were than classified into the following training modalities:

- Change of direction technique modification training: COD drills performed with coach feedback and cues that focus on modifying COD technique, such as lateral foot plant distance/ trunk positioning.
- COD speed and footwork training: pre-planned COD drills with no coach feedback and cues regarding COD technique.
- Balance training: balance training that incorporates stable and unstable training methods, such as balancing on one leg (while catching a ball), wobble boards, etc.
- Mixed training programmes: sessions that integrate exercises from at least three or more of the following training modalities: plyometrics, stretching, balance, trunk stabilisation and/or resistance training. These involve dedicated sessions performed outside typical sports-specific practice and games.
- Resistance training: sessions that include free weight and/or machine-based resistance training.
- Perturbation-enhanced plyometrics: plyometric training performed with added perturbation (motorised platform) over weight acceptance.
- Trunk stabilisation training or dynamic core stability training: trunk stabilisation training refers to training with static exercises (i.e. planks, etc.). Dynamic core stability training includes exercises performed dynamically (i.e. dynamic planks, bridges, etc.) with added perturbations.
- Combined training: training that combines two of the abovementioned training modalities. These are sub-divided into: combined COD technique modification and balance training, combined trunk stabilisation and resistance training, and combined resistance training and intersegmental control training during running and COD drills.
- Warm-up interventions: neuromuscular warm-up interventions that were typically performed 15–25 minutes prior to sport-specific practice (i.e. technical and tactical) and/or games. These warm-ups replaced their normal skill/tactical warm-up, and include exercises from various training modalities, such as trunk stabilisation, plyometrics, balance, body weight resistance training, running and COD drills. These include the Oslo Neuromuscular warm-up, core position and control (Core-Pac) warm-up intervention, and FIFA’s Medical Assessment and Research Centre 11+ (F-MARC 11+) soccer-specific injury-prevention warm-up.

The following sections outline the findings of included studies relevant to the effects of specific training interventions on COD biomechanics associated with ACL loading.

## Results

Initial database searches resulted in the identification of 1,021 articles, with an additional six articles through bibliographies, citation tracking and hand searching (Fig. 1). After removing duplicates, 928 articles were retained for initial screening. Title and abstract screening resulted in 889 articles excluded. The remaining 29 articles were further examined using the inclusion/exclusion criteria, and seven studies were excluded, resulting in 25 datasets from 22 studies included to examine the effect of training intervention on COD biomechanics associated with increased ACL loading (Fig. 1 and Tables 1, 2, 3, 4).

**Table 1:** Summary of research that has investigated the effects of COD technique modification and COD speed and footwork training on COD biomechanics

Study	Subjects	Training intervention	COD task	Results (post-intervention)	Comments
*COD technique modification drills*					
Dempsey et al. [22]	Twelve male non-elite team sport (6 Australian football, 5 rugby union, and 1 soccer) athletes *3 withdrawals	6-week COD technique modification 2 × a week (15-min sessions) With immediate feedback (visual and oral)	45° ± 5° side-step. PP and UP ~5 m.s^−1^	At IC: **↓ lateral foot plant distance** (*p* = 0.039) PP (ES = 0.55), UP (ES = 0.58) **↓ lateral trunk flexion** (*p* = 0.005) PP (ES = 1.09), UP (ES = 0.14) At WA: ↓ peak **KAM** (*p *= 0.034) PP (ES = 0.58) and UP (ES = 0.78) Both postural changes were correlated with the change in KAM **change in lateral foot plant distance** (*r* = –0.468, *p = *0.025) **lateral trunk flexion** (*r* = − 0.377, *p* = 0.135) ↔ in approach speed, knee flexion IC, and torso rotation	No CG Did not establish reliability, measurement error, or meaningful difference Implications on performance unclear Controlled approach velocity
Jones et al. [56]	Ten female netball players	6-week COD technique modification 2 × a week Technique drills that encouraged PFC braking, backwards trunk inclination, and neutral foot position. Weeks: 1 and 2—deceleration emphasis; 3 and 4—randomly with greater entry velocity; 5 and 6—drills performed randomly at speed unanticipated stimulus	180° turn –PP ~3 m.s^−1^	↓ **completion time** (*p* < 0.05, ES = 0.74) ↓ **peak KAM** (*p* < 0.001, ES = 0.73) ↓ i**nitial foot progression angle** (*p* < 0.001, ES = 2.60) **↓ initial trunk angle at FFC** (*p* < 0.05, ES = 0.58) **↔** in approach velocity or horizontal GRF ratio (ES = 0.10–0.15) **Changes in initial foot progression angle and KAM** (*r*^*2*^ = 37%, *p* = 0.028)	Athletes were not fast to begin with No CG Did not establish reliability, measurement error, or meaningful difference Conference proceeding format
*Change of direction speed and footwork*					
Wilderman et al. [57]	30 female basketball players	6-week agility (COD speed, footwork, and manoeuvrability drills)—4 × a week (*N* = 15) plus a CG (*N* = 15)	45° side-step Sidesteps—PP 3.3–4.3 m.s^−1^	**↑ medial hamstring EMG activation** for IG (ES = 0.94) ↔ in knee flexion angle and vertical GRF (*p* > 0.05, ES ≤ 0.15)	Lack of feedback regarding COD technique Absence of specific side-stepping drills

↑ increase, ↓ decrease, ↔ no significant change, *KAM* knee abduction moment, *IC* initial contact, *WA* weight acceptance, *IRM* internal rotation moment; *ROM* range of motion, *GCT* ground contact time, *BW* body weight, *GRF* ground reaction force, *PP* pre-planned, *UP* unplanned, *BW* body weight, *EMG* electromyography, *PFC* penultimate foot contact, *FFC* final foot contact, *ES* effect size, *CG* control group, *IG* intervention group, *COD* change of direction, *GRF* ground reaction force

**Table 2:** Summary of research that has investigated the effects of balance, dynamic core stability control training and perturbation-enhanced plyometric training on COD biomechanics

Study	Subjects	Training intervention	COD task	Results (post-intervention)	Comments
*Balance training*					
Oliveira et al. [67]	26 healthy men—recreational athletes	6-week balance training—4 × a week (30 mins) (*n* = 13) plus a CG (*n* = 13)	90° cut and 1 unexpected perturbed cut (10 cm translation) ~ 2.5 m.s^−1^	Balance group during perturbed cutting ↓ **peak KAMs** (33 ± 25%, *p* < 0.03, η^2^ = 0.487) ↑ **activation of trunk and proximal hip muscles** ↑ **burst duration** prior (23 ± 11%) to landing (*p* < 0.02, η^2^ = 0.798) ↔ changes in peak force, approach and exit velocity (*p* < 0.05)	Presents findings for the perturbed trial only, and this was for only 1 trial Low approach velocity
Cochrane et al. [68]	Fifty male AFL players	Allocated either to a CG or to one of four 12-wk training programs: Machine weights Free weights Balance Machine weights and balances	30˚ and 60˚ side-step, 30˚ XOC PP and UP and—light delay ~4–4.5 m.s^−1^ Preferred leg	Balance group **↑ flexor/extensor contraction ratio** − 18% **↑ flexor muscle activation** **↑ biceps femoris/semimembranosus co-contraction ratio** **↓ quadricep activation** Strength training **↓ flexor/extensor contraction ratio** and ↑ q**uadricep activation**	Implications on performance unclear Controlled approach velocity
Cochrane et al. [63]	Fifty male AFL players	Allocated either to a CG or to one of four 12-wk training programs: Machine weights Free weights Balance Machine weights and balance	30˚ and 60˚ side-step, 30˚ XOC PP and UP and—light delay ~4–4.5 m.s^−1^ Preferred leg	Change in moments across WA in all manoeuvres (Mean and SD not provided, thus ES cannot be calculated): **Balance** **↓ peak KAM** (*p* < 0.001, 62%) and **↓ peak IRM** (*p* < 0.001, 32%) in all manoeuvres **Free weights** ↔ peak KAM and IRM **Machine Weights** ↓ **peak KAM** (*p* < 0.05, 27%) **Machine weights + balance training** ↔ peak KAM and IRM **CG** ↑ **peak KAM** (*p* < 0.05, 26%)	Did not establish reliability, measurement error or meaningful difference Implications on performance unclear Controlled approach velocity
*Dynamic core stability training*					
Whyte et al. [66]	31 male varsity footballers	6-week dynamic trunk control/core stability programme—3 × a week (*n* = 15) plus a CG (*n* = 16)	45° side-step PP and UP	**IG** **↑ internal hip extensor moment** (*p* = 0.017, *η*^2^ = 0.079,24–28% of stance) for PP **↓ internal knee varus moment** (*p* = 0.026, *η*^2^ = 0.076, 18– 25% of stance) for PP **↓ knee external rotator moment** (*p* = 0.041, *η*^2^ = 0.066, 15– 20% of stance) for PP **↓ posterior GRF** for both cuts (*p* ≤ 0.030, *η*^2^ = 0.074–0.081) for PP and UP (11–30% and 15–19% of stance, respectively) ↔ **in trunk and pelvic kinematics** (Descriptive data not provided, thus ES cannot be calculated)	Use of SPM Contains CG
*Perturbation-enhanced plyometric training*					
Weltin et al. [69]	28 females (soccer, handball, and basketball)* 4 withdrawals:	Perturbation-enhanced plyometric training (PPT) (n = 12): lateral reactive jumps—4-week training—3 times a week Plyometric only—CG (*N* = 12)	45° side-step UP—4.0 ± 0.2 m.s^−1^	**PPT** **↓ trunk rotation** 7.2° (ES = 1.14), **↓ step width** (*p* = 0.003, ES = 0.88), and **↑ pelvic rotation** 4.1° (ES = 0.45) **↓ KAM** 0.05 Nm/Kg, CG **↑ 0.14** Nm/kg (SD not provide, thus ES cannot be calculated) ↔ lateral trunk lean (ES = 0.26)	Perturbation-enhanced method is unfeasible to implement in real world as it required motored platform

↑ increase, ↓ decrease, ↔ no significant change, *GRF* ground reaction force, *PP* pre-planned, *UP* unplanned, *ES* effect size, *CG* control group, *IG* intervention group, *SPM* statistical parametric mapping, *PPT* perturbation and plyometric training, *KAM* knee abduction moment, *IRM* internal rotation moment, *XOC* crossover cut

**Table 3:** Summary of research that has investigated the effects of a mixed training programmes and combined programmes on COD biomechanics

Study	Subjects	Training intervention	COD task	Results (post-intervention)	Comments
*Mixed programme—session that integrates exercises of at least three of the following modalities: trunk, balance, plyometric, strength training, flexibility*					
Weir et al. [58]	10 elite female hockey players	16-week maintenance training program (3 × 10-min sessions a week) which directly followed a high-dose 9-week initial training intervention (4 × 20-min sessions a week), as part of a biomechanically informed ACL injury prevention program BW plyometric, resistance, and balance exercises	45˚ side-step—UP	↓ **peak KAMs** (ES = 0.30, 26.3%) following maintenance High-risk responders displayed **↓ peak KAM** (28.6%) and **IRM** (37.1%)	Highlight the importance of continuing the training Acknowledged there will be responders and non-responder Abstract format
Weir et al. [61]	13 elite female hockey players	8-week 4 × 15-min multi-component sessions consisting of BW plyometric, resistance, and balance exercises	45˚ side-step—UP	↑ TMA of the gluteal (grouped maximus and medius) by 10% during WA (*p* = 0.006, power = 0.864). ↔ in frontal plane knee moments (*p* < 0.01, ES = 0.73), ↑ hip extension moment (ES = 0.56)	No differences in frontal plane knee moments Abstract format No CG
Yang et al. [60]	22 male, 18 female (basketball and volleyball)	4-week multi-component programme consisting trunk strengthening, stretching, proprioceptive training, hip extension strength training and plyometric training intervention—3 × a week (N = 18, 9 male and 9 female) Plus a CG (N = 18, 9 male and 9 female)	45° side-step Sidesteps—PP—5 step run-up	↔ no differences in knee flexion angles, peak impact posterior GRF, or exit velocities compared to CG following intervention (4–12 weeks post-training intervention)	Multicomponent programme; however, strength exercises were prescribed for, strength/ muscular endurance Considered only limited number of variables—unknown the effect of frontal plane biomechanics Short duration
Bencke et al. [59]	17 male handball players	Mixed programme consisting of jump landings, unilateral squats, hamstring pulls, hip abductions, and one-leg coordinated hopping IG (n = 10) 12 weeks twice a week Plus a CG (n = 7)	Side-step (no other decsriptions provided)	IG **↑ VPF** (ES = 0.41), **↓ GCTs** (*p* < 0.05, ES = 0.94) due to a ↓ concentric phase duration (*p* < 0.05, ES = 0.94) **↓ ST** (*p* < 0.05, ES = 0.63) and **BF** pre-activity duration (*p* = 0.08, ES = 0.59)	No joint kinetics/kinematics examined
Staynor et al. [53]	25 female community-level team sport athletes *6 withdrawals for training group	Split into IG (*n* = 8) and CG (*n* = 10), 2 × a week for 9 weeks (15- to 20-min sessions) Combination of BW plyometric, resistance, and balance exercises	Side-step—UP (full details not provided)	IG **↑ peak KFM (ES = 0.77),** ↔ in **peak KAM (ES = 0.16)** and **IRM (ES = 0.0)**, but **CG ↑ peak KAM** (ES = 0.36, 28%) and **↑ IRM** (ES = 0.56, 38%) IG **↓ hip abduction** (ES = 0.70, 31%) **↑ knee flexion at foot strike** (ES = 0.59, 33%) **↓ trunk flexion range of motion** (ES = 0.97, 29%) **↓ lateral trunk flexion** (ES = 0.40, 16%) **↓ lateral foot plant distance** (ES = 0.84, 11%)	Did not establish reliability, measurement error, or meaningful difference Attendance and compliance rates of 71 ± 14 and 77 ± 7%
*Combined balance and COD technique training*					
Donnelly et al. [64]	AFL male athletes (*n* = 1001) 34 athletes for biomechanical testing (BTT, *n* = 20; ST, *n* = 14)	Balance and COD technique training (BTT) or acceleration training (ST) 2 × week—20 min/week—18 weeks 1 × week—weeks 19–28	45° ± 5°, side-step Sidesteps—PP and UP	Both training groups: **↓ peak IRM** (*p* = 0.025, ES = 0.57)—45% reduction, during PP **↑ peak KAM** (*p* = 0.022, ES = 0.44)—31% increase during UP	High athlete to coach ratio (40:1) Low athlete compliance (45 ± 22%)
*Combined trunk stabilisation and resistance training*					
Jamison et al. [62]	22 males (previously played American football) N of 10 and 11 completed testing	RT only or Resistance and trunk stabilisation (TS) 6 weeks—3 sessions a week	45° ± 5°, side-step 3 steps self-selected jog	**RT only** **↑ peak KAMs** (*p* = 0.012, 50%) and **↑ peak IRM** (*p* = 0.617, 12%) **Combined training** **↑ peak KAM (***p* = 0.116, 35%) and **↓ peak IRM** (*p* = 0.110, 35%) (SD not provided, thus ES cannot be calculated)	Did not achieve *a priori* minimum sample size recommendations Did not establish reliability, measurement error, or meaningful difference Static trunk exercises were used
*Combined resistance training, and intersegmental control training during running and COD drills*					
King et al. [65]	112 athletes with athletic groin pain were assessed pre- and post- rehabilitation	Athletes were subjected to three levels of rehabilitation: Level 1 intersegmental control and strength training Level 2 linear running drills (lumbo-pelvic control and posture) Level 3 multidirectional technique drills that emphasised segmental control (using holding a medball, or arms locked overhead) and lateral propulsion	110° cut -PP, ~ 2 m.s^−1^	**↓ ipsilateral trunk side flexion** (ES = 0.79) **↓ hip abduction angle and hip adduction moment** **↑ pelvic rotation** in the direction of travel (ES = 0.76) **↑ centre of mass translation** in the direction of travel relative to centre of pressure (ES = 0.40) **↓ knee flexion angle** (ES = 0.33) **↑ ankle plantar flexor moment** (ES = 0.48) ↔ in approach velocity (*p* = 0.434, ES = 0.07) ↓ **GCT** (ES = 0.30) ↑ **dorsi-flexion** (ES = 0.58) Large increase in total work done at the ankle, a moderate reduction in the total work done at the hip, and a small reduction at the knee after rehabilitation.	Considered performance implications Showed positive effects for injury risk and performance No CG Did not establish reliability, measurement error, or meaningful difference

↑ increase, ↓ decrease, ↔ no significant change, *XOC* crossover cut, *KAM* knee abduction moment, *WA* weight acceptance, *IRM* internal rotation moment, *SD* standard deviation, *BW* body weight, *GRF* ground reaction force, *PP* pre-planned, *UP* unplanned, *BW* body weight, *ACL* anterior cruciate ligament, *RT* resistance training, *ES* effect size, *CG* control group, *TMA* total muscle activation, *CG* control group, *COD* change of direction, *VL* vastus lateralis, *BF* biceps femoris, *ST* semitendinosus, *VPF* vertical propulsive force

**Table 4:** Summary of research that has investigated the effects of injury prevention warm-up protocols on COD biomechanics

Study	Subjects	Training intervention	COD task	Results (post-intervention)	Comments
*F-MARC 11+ soccer specific warm-up*					
Thompson et al. [70]	51 females aged 10–12 years soccer players *5 withdrawals	F-MARC 11 + (*n* = 26) 2 × a week for 7–8 weeks—15 sessions total plus CG (*n* = 20)	45° ± 5°, side-step Sidesteps -PP and UP ~ 4 m.s^−1^	**Bilateral jump (IG)** **↓ peak KAM** (*p* = 0.045, ES = 2.15) **Side-stepping (IG)** **↑ peak KAM** PP (*p* = 0.280, ES = 1.20, 10%) and UP (*p* = 0.044, ES = 1.98, 18%)	Did not establish reliability, measurement error, or meaningful difference Athlete compliance 70.2 ± 14.0%
Thompson-Kolaser et al. [71]	51 preadolescent females (28 intervention, 23 CG)* 5 withdrawals and 43 adolescent (22 intervention, 21 CG)* 6 withdrawals	F MARC 11 + (*n* = 26) 2 × a week for 7–8 weeks—15 sessions total plus CG (*n* = 20)	45° ± 5°, side-step Sidesteps -PP and UP ~ 4 m.s^−1^	**Preadolescents—PP side-step** **↑ precontact flexor-extensor muscle contraction** (*p* = 0.004-0.002) **Both groups—side-step** ↔ in **knee valgus angles** or peak **KAM**—Inspection of graphs indicate ↑ peak KAMs in both groups (Descriptive data not provided so ES cannot be calculated)	Highlights ineffectiveness of intervention for addressing cutting mechanics—only effective for bilateral task Lack of volume and exercises that addresses COD mechanics with feedback, and lack of dynamic trunk exercises
*Oslo neuromuscular injury-prevention warm-up*					
Zebis et al. [73]	Elite handball (*n* = 8) and elite soccer (*n* = 12) players	Oslo NMS warm intervention—20 min warm up—one season	Side-step (no other decsriptions provided)	**↑ Pre-landing EMG activity ST** (*p* < 0.001, ES = 0.70-0.78) and activity at foot strike (*p* < 0.05, ES = 0.60) ↔ **Quadriceps EMG** (ES = 0.10-0.23) ↔ **Knee and hip joint angles** (ES = 0.11)	Low sample size Investigated low number of biomechanical variables No CG
Zebis et al. [72]	40 adolescent female football and handball players	12 week Oslo NMS warm up—3 × a week (*n* = 20) plus a CG (*n* = 20)	Side-step (no other decsriptions provided)	IG **↓ VL-ST activity difference** (43%, *p* < 0.0001) **↑ hamstring MVC** (*p* = 0.0134) **↓ VL EMG preactivity** (23%, *p* < 0.0008), **↑ ST EMG preactivity** (18%, *p* < 0.0001), and **↑ BF EMG preactivity** vs CG ↔ **peak KAM** or **knee valgus angle at IC** (Descriptive data not provided, thus ES cannot be calculated)	Only frontal plane knee kinetics and kinematics
*Core-Pac warm-up*					
Celebrini et al. [75]	Ten adolescent female soccer players	baseline testing—acute changes (*n* = 10) (move from the centre- lead with the belly button) 4 week—Core-Pac training intervention (*n* = 7) 4 × week	15–55° side-step PP and UP	5 of 7 subjects displayed **↑ knee flexion angle and ↓ peak KAM**	Individual differences in response to training intervention No CG
Celebrini et al. [74]	Twenty adolescent female soccer players	6 week—Core-Pac training intervention—4 × a week (*n* = 10) plus a CG (*n* = 9)	15–55° side-step PP and UP	IG **↑ knee flexion angle** PP cutting (*p* = 0.001, ES = 2.02) ↔ **peak KAM** for PP and UP (Raw data not provided, thus ES cannot be calculated)	Low sample size No immediate feedback regarding their technique or biofeedback

↑ increase, ↓ decrease, ↔ no significant change, *KAM* knee abduction moment, *IC* initial contact, *IRM* internal rotation moment, *GCT* ground contact time, *BW* body weight, *NMS* neuromuscular, *PP* pre-planned, *UP* unplanned, *EMG* electromyography, *RT* resistance training, *ES* effect size, *CG* control group, *IG* intervention group, *COD* change of direction, *SD* standard deviation, *VL* vastus lateralis, *BF* biceps femoris, *ST* semitendinosus, *VPF* vertical propulsive force, *MVC* maximal voluntary contraction, *Core-Pac* core position and control, *F MARC 11 +* FIFA NMS warm-up, *IC* initial contact

Two studies investigated the effects of COD technique modification training [22, 56], while one study examined the effects of COD speed and footwork training [57]. Five studies examined the effects of a mixed training programme [53, 58–61], while one study investigated the effects of combined COD technique modification and balance training [64], combined trunk stabilisation and resistance training [62], and combined resistance training and intersegmental control training during running and COD drills [65]. Three studies examined the effects of balance training [63, 67, 68], two studies examined resistance training [62, 63], while one study examined the effects of dynamic core-stability training [66], and one other study examined perturbation-enhanced plyometric training [69]. Two studies examined the effects of the Oslo neuromuscular warm-up protocol [72, 73], two studies examined the core position and control (Core-Pac) warm-up intervention [74, 75], and two studies examined FIFA’s Medical Assessment and Research Centre 11+ (F-MARC 11+) soccer-specific injury-prevention warm-up [70, 71] interventions on COD biomechanics. Eleven of the 22 studies failed to include a control group (Tables 1, 2, 3, 4). Only one study provided reliability measures for biomechanical variables, but no study acknowledged measurement error or established smallest worthwhile change or smallest detectable difference when interpreting findings (Tables 1, 2, 3, 4). The effects of these training interventions on COD biomechanics are presented in Tables 1, 2, 3, 4.

## Discussion

The primary purpose of this scoping review was to critically appraise and comprehensively synthesise the existing literature related to the effects of training interventions on COD biomechanics associated with increased knee joint loads and subsequent ACL loading, and identify gaps in the literature with subsequent recommended areas for further research. The primary findings were balance and COD technique modification training appear to be the most effective training modalities for reducing knee joint loading (small to moderate effect sizes) during COD while other training modalities were generally ineffective (Tables 1, 2, 3, 4). Although the published literature regarding the effectiveness of training interventions on COD biomechanics associated with increased ACL loading is indeed insightful, there are key methodological and research design limitations that must be acknowledged going forward to improve our understanding of effective training strategies that reduce COD knee joint loads. These limitations include, in general, small sample sizes (18 studies *n* = 7–20 for intervention group), lack of control groups (11 studies contained no control group), failure to establish reliability measures (21 studies) and acknowledging measurement error to establish real and meaningful changes, and generally failing to consider the implications on performance. The effectiveness of the different training modalities, gaps in the literature, and recommended areas of further research are discussed in more detail below.

### Change-of-Direction (COD) Technique Modification Training

In order to reduce knee joint moments and subsequent ACL loading, the magnitude of the GRF or the moment arm must be reduced [23]. Several studies have shown that acute (within-session) changes in COD technique can reduce knee joint loads [21, 75, 77], such as narrowing lateral foot plant distance and changing trunk orientation [21], increasing knee flexion [77], and moving the centre of mass closer to the base of support [75]. Because of the promising results observed with acute COD technique modification, several studies have investigated the chronic effects of COD technique modification on COD biomechanics associated with increased ACL loading [22, 56] (Table 1).

Dempsey et al. [21] initially examined the effects of an acute within-session COD technique modification (altering foot plant distances, trunk positioning and foot orientations) on 45˚ side-step biomechanics. A wide foot plant combined with lateral trunk flexion over the plant foot resulted in the greatest peak KAMs (*p* ≤ 0.003, ES = 0.75–0.97), while a wide foot plant with torso rotation towards the plant foot resulted in significantly (*p* = 0.001, ES = 1.00) greater peak IRMs. These findings are concerning because knee frontal and transverse moments can increase ACL strain [25, 37–39]. Conversely, a side-step technique that involved neutral foot positioning, a foot plant distance closer to the midline, and an upright (in frontal plane) torso resulted in the lowest knee joint loading (KAM and IRM), due to reducing the moment arm between the GRF and knee joint centre [21]. As such, a narrow foot placement with an upright trunk was subsequently advocated as a safer side-stepping technique [21].

Expanding on the promising results of the acute side-stepping technical modification, Dempsey et al. [22] investigated the effects of a 45° side-stepping technique modification intervention over 6 weeks (2 × 15 mins sessions per week) on COD biomechanics (Table 1). The intervention consisted of performing side-step drills with imposed technique changes by bringing the foot closer to the midline (tape placed on floor for acceptable foot plant distance), maintaining an upright torso, and having the torso facing towards the direction of travel. Importantly, participants were provided with oral and video feedback regarding their technique between repetitions. The authors, notably, demonstrated significantly lower peak KAMs (*p * = 0.034, ES = 0.58–0.78, 36%) during both anticipated and unanticipated side-step tasks accompanied with significant reductions in lateral foot plant distance and lateral trunk flexion (*p* ≤ 0.039, ES = 0.14–1.09) (Table 1). As such, side-step technique modifications were effective in reducing knee joint loading, and in turn, could be an effective strategy to reduce non-contact ACL injury-risk.

Although the acute [21] and chronic COD technique modifications [22] by Dempsey et al. have shown positive reductions in knee joint loading during directional changes, a note of caution is warranted. Firstly, the abovementioned studies have failed to present and acknowledge measurement error values; thus, it is uncertain whether such changes were greater than the measurement error, and therefore real. Secondly, the training intervention performed by Dempsey et al. [22] did not contain a control group; therefore, the results should be interpreted with caution. Although reducing lateral foot plant distance was shown to reduce peak KAMs [22], critically, this imposed technique change could be detrimental for medio-lateral force application and may result in suboptimal COD performance (i.e. reduced exit velocity from the push-off) [31, 32, 78, 79]. It is worth noting, however, athletes adopted less lateral trunk flexion (i.e. more upright trunk), which may be a positive adaptation for faster cutting performance [80]. Moreover, the studies performed by Dempsey et al. [21, 22] have failed to consider the implications of such changes in side-step technique on COD performance (i.e. ground contact time [GCT], COD exit velocity and completion time). As athletes are driven by performance, they may be unlikely to adopt movement techniques that decrease the risk of knee injury if they do not result in effective performance [32]. Consequently, further research is necessary investigating the chronic effects of side-stepping technique modification on both biomechanics associated with decreased ACL loading and increased performance [41].

Investigating a sharper COD (180°), Jones et al. [56] reported a reduction in turning KAMs (ES = 0.73) and improved completion times (ES = 0.74) in female netball players as a result of a 6-week technique modification intervention that consisted of technical drills that encouraged penultimate foot contact braking, backwards trunk inclination and neutral foot positioning (Table 1). Interestingly, a strong association between changes in initial foot progression angle and KAMs (*r*^*2*^ = 37%, *p* = 0.028) was observed, while athletes also demonstrated changes in trunk inclination during the final foot contact (ES = 0.58). However, similar to Dempsey et al. [22], there was no control group, and findings were not interpreted in relation to the measurement error. Nevertheless, instructing athletes to adopt a more neutral foot progression angle (i.e. closer to 0°) during sharper 180° turns could be an effective strategy to reduce peak KAMs and subsequent ACL loading.

Collectively, COD technique modification appears to be a potentially viable and effective strategy in reducing knee joint loading (Table 1); however, published COD technique training interventions lack control groups and do not acknowledge measurement error when interpreting findings. Moreover, it is unknown how long such changes in COD biomechanics are retained following a training intervention. Thus, further COD technique modification interventions are required that include a control group and acknowledge measurement errors to definitively confirm the effectiveness of this training modality in reducing knee joint loading. Moreover, COD performance should also be considered to understand the implications of such technical modifications on knee joint loading and performance because athletes may be unlikely to adopt safer strategies at the expense of performance. If COD performance can be maintained or improved while simultaneously reducing knee joint loading following COD technique  modification, this would help improve adherence and may provide practitioners with an effective strategy to mitigate injury risk.

### COD Speed and Footwork Training

Wilderman et al. [57] examined the effects of a 6-week agility training programme that was performed four times a week by female basketball players compared to a control group. The programme consisted of pre-planned COD speed, footwork and manoeuvrability drills; thus, the term “agility” is incorrect due to the absence of drills that involve responding to an external stimulus [81, 82]. Nevertheless, the intervention group showed increases in medial hamstring activation (ES = 0.94) (Table 1), which may help reduce anterior tibial shear and subsequent ACL strain [83–87], though no statistically significant (ES ≤ 0.15) changes in knee flexion angle or vertical GRFs were observed. A limitation of this study was the lack of specific drills that focused on side-stepping mechanics. In addition, the absence of coach feedback regarding the athlete’s technique is also a limitation that may explain the mixed results. Conversely, studies that have documented positive changes in COD technique [22, 56] have emphasised the importance of coach technical feedback. It is also worth noting that the biomechanical variables examined during the side-step by Wilderman et al. [57] were limited to only knee flexion angle, GRF and muscle activity; thus, a more comprehensive analysis of frontal plane biomechanics and trunk kinematics would have strengthened this study, because these factors are strong determinants of knee joint loading [21, 23, 41].

### Balance Training

Because lower-limb balance training has been shown to be effective in reducing ACL injury rates in sport [88, 89], several studies have attempted to identify the underlying biomechanical and neuromuscular mechanisms that may explain the reductions in ACL rates (Table 2). Oliveira et al. [67] demonstrated 6 weeks of balance training resulted in a statistically significant 33% reduction in peak KAMs during a perturbed cutting task, while a control group demonstrated a slight increase, though not statistically significant (Table 2). The improvement in peak KAMs was accompanied with increased EMG activation of the trunk and proximal hip musculature and increased EMG burst duration prior to initial contact (Table 2). Although trunk kinematics were not examined, the authors hypothesised the improved muscle activity of the hip and trunk lead to improvements in trunk control, which is a critical factor for knee joint loading [21, 31, 33, 34]. It is worth noting, however, that pre- and post-analysis in perturbed cutting biomechanics and muscle activation was only performed for one trial. This is a problematic issue because evaluations based only on one trial can lead to invalid data and erroneous conclusions [90, 91], while one trial may not be fully representative of an athlete’s typical movement pattern [91].

Reporting a similar finding to Olivera et al. [67], but investigating a greater trial size, Cochrane et al. [63] found balance training was the most effective modality to reduce both peak KAMs (*p* < 0.001, 62%) and peak IRMs (*p* < 0.001, 32%) in all anticipated and unanticipated COD manoeuvres (Table 2), compared to machine-based resistance training, free weight and combined machine-based and balance training. While machine-based training was also effective in reducing peak KAMs (*p* < 0.05, 27%), free weight and combined machine-based weights and balance training were ineffective in reducing KAMs or IRMs (Table 2), and a control group increased their peak KAM. The reductions in frontal and transverse plane joint loads as a result of balance training may be explained by earlier work from Cochrane et al. [68], who that found 12 weeks’ balance training elicited positive and potentially safer changes in lower-limb muscle activation. Increased knee flexor/extensor contraction ratios, increased flexor muscle activation, and increased biceps femoris/semimembranosus contraction ratios were observed, while a strength training group increased their quadriceps activation and reduced their hamstring activation (Table 2). The hamstrings are considered to have an important role during the weight-acceptance phase of COD in preventing anterior tibial translation and reducing anterior tibial shear and ACL strain [55, 83–87].

Consequently, the results from these studies suggest that balance training could be an effective training modality for reducing COD knee joint loading (Table 2) and subsequent ACL loading. The successful results are most likely attributed to positive changes in hamstring, hip and trunk muscle activation, which supports and reduces knee joint loading [55]. It is worth noting, however, that the aforementioned studies failed to acknowledge measurement error when interpreting their findings and did not consider the performance implications; thus, is a future direction of research to definitively conclude the effectiveness of this method. Nevertheless, balance training involves the use of wobble boards, instability surfaces and catching a ball, which is easy to perform, simple to regress and progress, and can be easily integrated into athletes training programmes to help reduce ACL loading and potential injury risk.

### Mixed Training Programmes

Several studies have used mixed training programmes (sessions that integrate exercises from several training modalities, i.e. plyometrics, stretching, balance, trunk stabilisation and/or resistance training) [53, 58–61] or a combination of training modalities in an attempt to alter COD biomechanics associated with increased ACL loading (Table 3).

#### Combination of Balance and COD Technique Modification Training

Based on the successful results of previous balance [63] and COD technique modification [22] interventions, Donnelly et al. [64] inspected the combined effects of balance training and COD technique modification compared to acceleration training on COD biomechanics. This intervention was performed in Australian Rules footballers (1,001 male athletes) over a regular season in a ‘real-world’ environment. Both training groups reduced their peak IRM during pre-planned side-steps (45% reduction), but peak KAMs significantly increased during unanticipated side-steps (31% increase) following the training intervention (Table 3), failing to substantiate the positive findings of previous research [22, 63]. Similar to previous COD technique modification and balance training interventions (Tables 1, 2), changes in knee joint loads were not interpreted in relation to the measurement error. The mixed findings of the training intervention by Donnelly et al. [64] could be explained by the low compliance rate of only 45% reported for the training intervention and a high athlete-to-coach ratio (40:1). These issues are problematic because successful training interventions that reduce knee joint load, thus ACL loading, are fundamentally underpinned by compliance [1, 22, 54, 63, 92–94]. Furthermore, the high athlete-to-coach ratios prevent sufficient biomechanical technique correction and feedback to individuals, which again limits the effectiveness of technique modification interventions [1, 22, 54, 92–95]. Additionally, a subset of only 34 athletes were examined for biomechanical testing throughout the season; thus, it is uncertain whether the subset’s biomechanics are fully representative of the whole sample (*n* = 1001).

Although balance [63] and COD technique modification [22] have been shown to be effective in reducing knee joint loading in controlled environments and in relatively small sample sizes (Tables 1, 2), the study by Donnelly et al. [64] highlights the potential difficulty in administering such training methods in ‘real-world’ environments at the community-level. The low adherence may be evident in such strategies to community-level athletes, who may not have the time or desire to complete further training outside typical sports practice, while the high athlete-to-coach ratio often associated at the amateur and community level makes it potentially unrealistic to provide individualised feedback. Therefore, these issues present a potential barrier in applying such strategies in the real world to attempt to reduce injury risk or investigate injury risk. Nevertheless, based on these findings, in order to perform a successful technique intervention that reduces knee joint loading, thus relative risk of injury, it is essential that there is high compliance and individual feedback regarding the athlete’s technique to facilitate effective changes in COD biomechanics [22, 54, 94].

#### Combination of Trunk Stabilisation and Resistance Training

Jamison et al. [62] compared the effects of combined resistance and trunk stabilisation (static trunk exercises) training compared to resistance training only on trunk control, strength and knee joint loading during a 45˚ unanticipated side-step. Significantly greater peak KAMs (*p* = 0.012, 50%) were observed for the resistance-training group only, and although not statistically different (*p* = 0.116), the combined group also displayed a 35% increase in side-stepping KAMs (Table 3). Conversely, the combined group demonstrated a 35% reduction in peak IRMs, though this was not statistically significant (*p* = 0.110), whereas IRMs increased 12% in the resistance training group (*p* = 0.617) (Table 3), though these changes were not interpreted in relation to the measurement error. Unsurprisingly, the combined group showed significantly greater improvements in core endurance and strength, while both groups improved 1RM deadlift strength (Table 3). This finding is similar to that of Cochrane et al. [63, 68], who also found resistance training was ineffective in reducing peak KAMs during a COD task, potentially due to the reduced hamstring and increased quadriceps activation, which may contribute to increased knee-joint loads [55]. Although performance measures were not examined (i.e. completion time, exit velocity, GCT) in the studies by Jamison et al. [62] and Cochrane et al. [63], the groups that performed resistance training increased their strength. Thus, it is speculated that the increased peak KAMs could be a by-product of an increase in approach velocity and an increased ability to produce force due to the strength training, both of which can influence knee joint loading [41, 48, 96].

Collectively, resistance training and combined resistance training and trunk stabilisation modalities appear ineffective in reducing COD knee joint loading (Table 3). The ineffectiveness of these training modalities, however, could be explained by the lack of task-specific training around trunk control and lower-limb control associated with multiplanar side-stepping [22, 66, 69]. Additionally, it should be noted that the trunk stabilisation intervention only included static exercises; however, dynamic trunk stabilisation exercises with perturbations may have provided a greater stimulus and specificity in order to reduce side-stepping knee joint loading [66, 69]. Furthermore, it is also worth acknowledging that a low sample size was investigated in the study by Jamison et al. [62] (n = 10 and 11), which failed to achieve adequate statistical power (a priori determined minimum sample of 18). It is must be noted, however, that although resistance training does not reduce knee joint loads during COD (Tables 2, 3), resistance training provides several benefits for athletes including enhanced performance during dynamic tasks (i.e. jumping, sprinting, COD) and positive adaptations to tissues (muscle, bone, ligament, tendon) [48, 97–99]. Moreover, as athletes become faster, improving their physical capacity through resistance training should enable them to tolerate the higher joint loadings [19, 23, 31, 55, 94, 97, 100, 101], thus highlighting the inclusion of resistance training in an athlete’s training programme.

#### Combined Resistance Training and Intersegmental Control Training During Running and COD Drills

King et al. [65] examined the effects of a rehabilitation programme that targeted intersegmental control in athletes with athletic groin pain. Athletes were subjected to three levels of rehabilitation: level 1 consisted of intersegmental control and strength training; level 2 focused on linear running drills focusing on lumbo-pelvic control and posture, and running mechanics; and level 3 focused on multidirectional technique drills that emphasised segmental control (holding a medball, or arms locked overhead) and lateral propulsion, which was performed three times a week. Repeat three-dimensional motion analysis revealed a 110˚ cutting task was performed with reductions in ipsilateral trunk side flexion (ES = 0.79), a factor linked to peak KAMs [21, 31, 33, 34], reduced hip abduction angle and hip adduction moment, which has also been linked to greater peak KAMs [29, 34, 49], and increased pelvic rotation in the direction of travel (ES = 0.76) (Table 3). Furthermore, changes in variables connected with faster cutting performance were revealed including greater COM translation in the direction of travel relative to centre of pressure (COP) (ES = 0.40), reduced knee flexion angle (ES = 0.33), and increased ankle plantar flexor moment (ES = 0.48). While no differences in approach velocity were observed (*p* = 0.434, ES = 0.07), a slightly shorter GCT was noted (ES = 0.30), indicating potential performance benefits [80, 102–104]. Unfortunately, KAMs or angles were not provided in the article, though it is speculated the positive changes in lateral trunk flexion, hip abduction and hip adduction moment may indicate a reduction in peak KAMs [29, 34, 49]. A note of caution is advocated, however, because there was no control group and measurement error values were not established.

#### Mixed Programme—Session Performed Separate from Sports Session that Integrates Exercises of at Least Three of the Following Modalities: Trunk, Balance, Plyometric, Strength Training, Flexibility

Yang et al. [60] recently examined the effects of a 4-week mixed-training intervention programme consisting of trunk strengthening, stretching, balance training, hip extension strength training and plyometrics in male and female basketball and volleyball players on 45° side-stepping. No statistically significant intervention effects on knee flexion angle, peak impact posterior GRF or exit velocities were observed (Table 3). As such, a 4-week mixed training intervention programme was ineffective in changing cutting biomechanics; however, 4 weeks could be a relatively short duration to potentially elicit positive adaptations, and it is worth noting that only three biomechanical variables were evaluated; thus, it is unknown what the effects were on frontal plane biomechanics, which are arguably of greater importance to injury risk [23, 41, 48]. Moreover, a note of caution is warranted for the hip-strengthening exercise repetitions prescribed by Yang et al. [60] because although the authors describe the protocol as strength training, the repetitions/durations prescribed were in fact strength endurance (30 s of one to two sets). This is sub-optimal for eliciting maximum strength adaptations where low repetitions with higher loads would be required [105, 106].

Bencke et al. [59] compared the effects a 12-week prophylactic training program on side-stepping GRF variables and muscle activity. The programme was performed twice a week, consisting of unilateral jump landings, unilateral squats, hamstring pulls, hip abductions and one-leg coordinated hopping in handball players in comparison to a control group who resumed normal skill training. Interestingly, the training intervention resulted in slightly greater vertical propulsive force (ES = 0.41), shorter GCTs (ES = 0.94) due to a shorter concentric phase duration (ES = 0.94), and a reduction in semi-tendinosis (ES = 0.63) and biceps femoris pre-activity duration (ES = 0.59) (Table 3). Therefore, the training programme had a positive effect on variables associated with faster COD speed performance such as greater vertical propulsive force [107, 108] and smaller GCTs [80, 102–104, 107], but the decreased hamstring muscle activity is of concern because high levels of hamstring muscle activation are needed to prevent anterior tibial translation and reduce anterior tibial shear [55, 83–87], thus ACL loading.

Weir et al. [61] demonstrated increases in total gluteal muscle activation and elevated contribution of hip extension moment to total support moment during unanticipated side-stepping following an 8-week mixed programme intervention (balance, plyometric and body-weight resistance training); however, no changes in frontal plane knee moments were observed in 13 female hockey players. Weir et al. [58] also demonstrated positive changes (reduced IRM) in unanticipated side-stepping biomechanics following a 9-week high-dosage mixed-training intervention (balance, plyometric and resistance training) (4 × 20-min sessions), but no statistically significant changes in frontal plane moments for the whole group were observed. Recently, Staynor et al. [53] examined the effects of a mixed programme training intervention, based on the intervention by Weir et al. [58] (consisting of plyometric, resistance and balance exercises, performed in-season twice a week for 9 weeks), on unanticipated side-stepping biomechanics in local female community-level athletes. Knee flexor moments increased post-training intervention (ES = 0.77), but no statistically significant changes in peak KAM and IRMs were observed for the training group (ES ≤ 0.16), whereas the control group displayed greater KAMs and IRMs (ES = 0.36–0.56) post-testing (Table 3). Additionally, the training group also produced kinematic changes associated with safer side-stepping cutting techniques such as reduced foot plant distances, more erect trunk postures in the frontal plane, and increased knee flexion (ES = 0.40–0.84, Table 3). It is worth noting, however, that all mixed training programme intervention studies have not acknowledged measurement error when interpreting their findings.

Consequently, based on the mixed training programmes intervention studies, it is inconclusive that this method of training is effective in reducing knee joint loading during COD. The results of these studies are in contrast to balance training [63, 67, 68] and COD technique modification interventions [22, 56], which have demonstrated reductions in COD knee joint loads. Although the mixed programmes did include balance exercises, the volume load and exercise duration of balances exercises were much lower than the successful interventions that solely focused on balance training. This discrepancy in volume load and duration may explain the contrasting findings. Additionally, it is speculated that the additional and combination of exercises from different modalities during these mixed programmes may interfere with balance training and may limit its effectiveness.

### Dynamic Core Stability Training

As the trunk contains over half of the body’s mass, deficits in neuromuscular control and suboptimal trunk motion and position is a critical factor affecting knee joint loading [109, 110]. Additionally, deficits in trunk control (i.e. core stability) have also been shown to be associated with non-contact ACL injury [111, 112]. Consequently, several studies have investigated the effects of trunk conditioning on COD biomechanics [62, 66]. Jamison et al. [62] reported what they defined as “combined resistance and trunk stabilisation” (which effectively involved solely static trunk exercises with resistance training) to be ineffective in reducing knee joint loads during cutting; however, in direct contrast, Whyte et al. [66] have recently demonstrated positive effects of a dynamic core stability intervention (i.e. trunk curls, dynamic bridges, planks, side planks, with added perturbations) on cutting mechanics (Table 2). Interestingly, following the 6-week intervention, athletes demonstrated increases in internal hip extensor moments and reductions in frontal and transverse knee joint loads (Table 2). This result is noteworthy because a combination of frontal and transverse knee joint loads can increase ACL loading to a greater extent than uniplanar loading [24, 28]. Additionally, reductions in posterior GRF were observed as a result of the training intervention. It is of note that this adaptation may result in reductions in anterior tibial shear [113], thus injury risk [37, 114–116]. Therefore, these findings indicate that dynamic core stability training could be an effective training modality to reduce ACL loading during cutting actions.

Surprisingly, trunk and pelvic kinematics remained unchanged following the intervention by Whyte et al. [66]; thus, the successful reductions in knee joint loads could be partially attributed to the reduction in posterior GRF. While this is a positive finding in terms of reducing potential ACL loading, the fact that posterior GRF was reduced may negatively affect performance, because posterior GRF has been associated with faster COD performance [102, 117, 118]. Unfortunately, Whyte et al. [66] did not examine cutting performance, but it is important to note that medio-lateral GRF will most likely be a larger contributing factor to faster cutting performance compared to posterior GRF [32, 78, 119], but this was not examined in the study. Future research needs to consider both injury risk and performance implications to improve our understanding of the potential performance-injury conflict present during COD.

The successful results of dynamic core stability training are in direct contrast to Jamison et al. [62]; however, these conflicting observations could be attributed to differences in exercise selection. For example, Jamison et al. [62] used static trunk stabilisation exercises, in contrast to the dynamic core stability exercises used by Whyte et al. [66]. The dynamic core stability exercises (with added perturbations) targets the centre of mass control and could be more specific to the trunk control requirements during cutting [69]. It should be noted, however, that only one study has confirmed that dynamic core stability training is effective in reducing knee joint loading during COD. Further research is required to definitively confirm that this training method is effective in reducing COD knee joint loads.

### Perturbation-Enhanced Plyometric Training

Weltin et al. [69] investigated the effects of perturbation-enhanced plyometric training (lateral reactive jumps on a motorised platform that moved) in comparison to regular plyometric training in female athletes. Interestingly, 4 weeks post intervention, the perturbation-enhanced plyometric group displayed reductions in trunk rotation and decreases in step width (Table 2), both of which are associated with greater KAMs [23, 34, 41, 48]. Although not statistically different, the perturbation-enhanced plyometric group showed a slight reduction in KAMs, while KAMs increased in the plyometric training group (Table 2). Surprisingly, lateral trunk lean remain unchanged following the perturbation-enhanced training; however, this absence could be attributed to the lack of feedback and cueing regarding trunk control in contrast to previous studies that have found positive changes in lateral trunk lean [22]. Consequently, perturbation-enhanced lateral reactive jump training reduces characteristics (trunk rotation and step width) associated with greater peak KAMs during directional changes but appears to be ineffective in producing statistically significant reductions in peak KAMs. Thus, more research is required around plyometric related interventions for the development of safer cutting mechanics.

### Injury-Prevention Warm-Up Training Protocols

Given the simplicity of training exercises to be integrated into the warm-ups of field-based sessions for athletes to improve neuromuscular control, and its relative success in reducing ACL injury rates [1, 89, 94, 120], several studies have investigated the effects of the Oslo, Core-Pac and F-MARC 11+ warm-up training interventions on COD biomechanics (Table 4). These interventions involve a 15- to 25-minute protocol that is performed prior to sport-specific practice (i.e. technical and tactical) and/or games.

#### Oslo Neuromuscular Warm-Up Intervention

Zebis et al. [73] found the Oslo warm-up training intervention increased pre-landing semitendinosus activity (*p* < 0.001, ES = 0.70–78), but unchanged quadriceps activity, hip and knee joint angles (ES = 0.10–0.23) during a side-stepping task in female handball and soccer players (Table 4). It is worth noting, however, that there was no control group, and only a limited number of biomechanical variables were examined (hip and knee joint angles, EMG activity). Including a control group, more recently Zebis et al. [72] examined the effects of the Oslo neuromuscular warm-up protocol on side-stepping biomechanics and EMG muscle activity. The intervention group displayed a potentially safer agonist-antagonist muscle pre-activity pattern, with elevated semitendinosus and biceps femoris pre-activity, and a reduction in vastus lateralis activity post-training, in contrast to the control group (Table 4). This finding is noteworthy because a lack of pre-activity observed with the medial hamstrings in combination with a greater proportion of lateral quadriceps recruitment may compress the lateral joint, open the medial joint, increase knee valgus, increase anterior shear force and therefore increase ACL loading [83–87]. For instance, in a cohort study, athletes who went on to injure their ACL displayed higher vastus lateralis pre-activity and reduced semitendinosus activity compared to uninjured athletes during a cut [121]. It is worth noting that Zebis et al. [72] observed no changes in peak KAM or knee valgus angles at initial contact following the training intervention, but unfortunately the authors failed to present the mean and standard deviations, thus the effect size could not be established. Consequently, the Oslo-warm-up protocol produces favourable agonist-antagonist muscle pre-activity patterns but appears to have a negligible effect on frontal plane knee moments.

#### Core-Pac Warm-Up Intervention

In light of the positive effects regarding the within-session changes in cutting technique adopting Core-Pac movement strategy [75], in the same study the authors also investigated the effects of Core-Pac warm-up training intervention in female soccer players. The warm-up consisted of balance, trunk, lower-limb control, multidirectional running, and COD drills. Due to a low sample size, statistical analysis was not performed; however, five of seven subjects displayed increases in knee flexion angle and reduced peak KAMs during cutting following the training intervention (Table 4). It is worth noting, however, that there was no control group, but the preliminary results highlight the individual variation in response to training interventions. Expanding on their previous work, Celebrini et al. [74] compared the effects of the Core-Pac warm-up in comparison to a control group who completed a normal warm-up routine. Following a 6-week intervention, the female soccer players who participated in the Core-Pac displayed an increased knee flexion angle during cutting (*p* = 0.001, ES = 2.02) (Table 4), but peak KAM remained unchanged. The researchers stressed two notes of caution: firstly, the study contained a low sample size and may therefore lack statistical power; and secondly, there was an absence of coaches’ and technological feedback regarding technique, which may explain the ineffectiveness in reducing peak KAM. The absence of feedback is in contrast to previous studies that have provided immediate feedback and subsequent successful reductions in knee joint loading [22, 56]. Consequently, further research is needed to confirm the efficacy of the Core-Pac training intervention on COD knee joint loading.

#### F-MARC 11+ Soccer-Specific Warm-Up

Thompson et al. [70] investigated the effects of the F-MARC 11+ soccer-specific warm-up on biomechanical risk factors associated with ACL loading in preadolescent female soccer players. The soccer players were divided into a control and an intervention group, with the neuromuscular warm-up performed twice a week for 7–8 weeks. Of concern, moderate to large increases (*p* ≤ 0.044, ES* = *1.18–1.95) in peak KAMs were demonstrated during pre-planned and unanticipated cutting (Table 4). Unfortunately, cutting performance was not examined, thus the implications of the F-MARC 11+ training intervention on performance is unclear. Critically, the F-MARC 11 + intervention was ineffective in reducing peak KAMs during side-step cutting. This finding is noteworthy because cutting actions are associated with non-contact ACL injury, particularly in soccer [18, 42, 44, 45, 122].

More recently, Thompson-Kolesar et al. (2018) has also confirmed that the F-MARC 11+ soccer-specific warm-up was ineffective in reducing peak KAMs or knee valgus angles during cutting tasks in adolescent athletes (Table 4), substantiating the results of their earlier study in preadolescents. This observation could be attributed to the lack of repetitions and volume of COD technique training in the programme. The F-MARC 11+ programme primarily consists of bilateral tasks such as squats and jump-landings that are integrated with balance and trunk conditioning, which could explain why KAMs reduced during the bilateral drop landing task only. Conversely, the technique modification intervention by Dempsey et al. [22] involved 15 minutes exclusively of COD technique modification, thus greater specificity and volume, resulting in reductions in KAMs. Therefore, these findings suggest the F-MARC 11+ does not adequately address deficits in cutting biomechanics in preadolescent and adolescent athletes but appears to be effective in reducing knee joint loading during bilateral landing activities.

### Maintenance Training

While reductions in biomechanical characteristics associated with ACL injury risk have been demonstrated with various training modalities [21, 22, 56, 58, 63, 77], it is also important to understand the training dosages required to retain the improved movement biomechanics and reduced knee joint loads following the training intervention. To the best of our knowledge, only one study has examined the effects of performing dosages of maintenance training following a period of high-dosage mixed training. Weir et al. [58] demonstrated positive changes (reduced IRM) in unanticipated side-stepping biomechanics following a 9-week high-dosage multicomponent training intervention (balance, plyometric and resistance training) (4 × 20-min sessions), and found a 16-week maintenance training programme (3 × 10-min sessions) resulted in meaningful reductions in peak KAM (-26.3%, *g = *0.30) (Table 3). As expected, the maintenance programme was particularly effective in retaining improved side-stepping biomechanics in the responder/high-risk group (classified as moderate-large effect size change) (Table 3). As stated previously, only one study has examined the effects of maintenance training dosages on COD biomechanics, thus, making it difficult to establish maintenance training guidelines. Consequently, more longitudinal studies are required that investigate the effects of maintenance training on COD biomechanics to improve our understanding regarding the maintenance of improvements in COD biomechanics.

## Conclusions

Based on the literature (Tables 1–4), balance training [63, 67, 68] is a potentially effective strategy to reduce knee joint loads during cutting; most likely attributed to eliciting safer knee agonist-antagonist muscle patterns and hip and trunk muscle activity. These positive biomechanical and neuromuscular adaptations may partially explain why balance training has been shown to reduce ACL injury rates [88, 89]. COD technique modification [21, 22, 56, 75, 77] also appears to be an effective training strategy for addressing COD biomechanical deficits associated with increased ACL loading and therefore potential non-contact ACL injury-risk. It should be noted, however, that the COD technique modification interventions that have shown promising results have not contained a control group and, as such, are a recommended area of further research. Moreover, the effectiveness of COD technique modification training on ACL injury rates has yet to be investigated. Nevertheless, in order to reduce knee joint moments and subsequent ACL loading, the magnitude of the GRF or moment arm must reduce [23]. As such, practitioners interested in reducing COD knee joint loading for their multidirectional athletes should consider incorporating balance and COD technique modification training into their athletes’ training programmes to reduce potentially hazardous knee joint loads when changing direction.

One study has shown promising results regarding the effectiveness of dynamic core stability training on COD knee joint loading [66], but further research is needed to definitively confirm the efficacy of this method. Perturbation-enhanced plyometric training [69], the F-MARC 11 + [70, 71], Oslo Neuromuscular warm-up protocol [72, 73] and resistance training [62, 63] are ineffective in reducing COD knee joint loads, whereas conflicting findings have been observed for the Core-Pac [74, 75], and mixed programme training interventions [53, 58–61, 65]. More research is required around plyometric-related interventions for the development of safer cutting mechanics. Although several studies have shown mixed training programmes and neuromuscular training appear to be ineffective in addressing COD biomechanics associated with increased ACL loading and potential non-contact injury risk (Tables 3 and 4), these training modalities have been shown to be effective in reducing ACL injury rates [1, 89, 94, 120] and may improve other qualities such as strength, muscle activation and athletic performance [1, 94]. Similarly, resistance training appears to be ineffective for reducing COD knee joint loads; however, this training modality elicits positive performance adaptations [97, 105, 123, 124] and is considered important for athletes to tolerate the loading associated when changing direction [19, 55, 94, 97, 100, 101]. Therefore, mixed training programmes, injury-prevention neuromuscular warm-ups and resistance training should not be overlooked, and warrant inclusion into an athlete’s holistic training programme.

Finally, to understand the most efficacious training modalities for addressing COD biomechanics associated with increased ACL loading, further research is needed in larger samples sizes, while containing a control group, and acknowledging measurement error to establish real and meaningful changes. Given the potential performance-injury conflict during COD [32, 41, 48], future studies need to consider the implications of the training intervention on both performance (completion time, GCT, exit velocity) and injury-risk biomechanics to better inform injury-risk mitigation programmes, because athletes may be unlikely to adhere to training programmes that negatively affect performance.
